# Comparison of the polyphenol content and *in vitro* antioxidant capacity of fruit-based nutritional supplements commonly consumed by athletic and recreationally active populations

**DOI:** 10.1080/15502783.2022.2091412

**Published:** 2022-07-04

**Authors:** Lee Rickards, Anthony Lynn, Margo E. Barker, Mark Russell, Mayur K. Ranchordas

**Affiliations:** a Sheffield United Footballl Club. Sport Science and Medical Department. Sheffield, UK; b Sport & Physical Activity Research Centre, Health Research Institute, Sheffield Hallam University, UK; c Food Group, College of Business, Technology & Engineering Sheffield Hallam University, Sheffield, UK; d Advanced Wellbeing Research Centre, Sheffield, UK; e School of Social and Health Sciences, Leeds Trinity University, Leeds, UK

**Keywords:** Antioxidants, anthocyanins, athletes, supplements, FRAP, fruit, cherry, blackcurrant, pomegranate, blueberry, ORAC, performance, polyphenols, nutritionists, recovery

## Abstract

**Background:**

Polyphenol-rich fruit supplements are commonly consumed by recreationally active and athletic populations because of their proposed benefits to both exercise performance and recovery from prior exercise. While it has been proposed that 300 mg of polyphenols pre-exercise enhances performance and 1000 mg per day accelerates recovery from muscle damage, it is difficult for consumers to optimize their intake because the polyphenol content of most fruit supplements is not available. Therefore, this study aimed to profile the phenolic and anthocyanin content and *in vitro* antioxidant capacity of a range of polyphenol-rich fruit supplements on sale in the UK.

**Methods:**

Ten polyphenol-rich fruit supplements (six cherry, two pomegranate, one blueberry, and one New Zealand blackcurrant) commonly consumed by athletes were analyzed for total phenols (Folin–Ciocalteu method), total anthocyanins (pH differential method), and *in vitro* antioxidant capacity (ferric reducing antioxidant power (FRAP) and oxygen radical absorbance capacity (ORAC).

**Results:**

The ten tested supplements varied markedly per serving in total phenolics (range: 13.8–1007.3 mg/gallic acid equivalents), anthocyanin content (range: 0.19–40.52 mg/cyanidin-3-glucoside), ORAC (range: 150–10,072 µmol of trolox equivalents), and FRAP (range: 72−14,320 µmol of Fe2+ equivalents). Different brands of tart cherry concentrate also exhibited a marked variation in their content of total phenolics (208–591 mg/GAE), anthocyanins (1.5–23.7 mg/cyd-3-glu), and antioxidant capacity (FRAP: 1724–4489 µmol of Fe2+ equivalents; ORAC: 6015–10,072 µmol of TE per serving) per serving.

**Conclusion:**

As expected, supplements based on different fruits contained different quantities of anthocyanins and polyphenols. However, there was also a substantial variation within different brands of tart cherry supplements. Because limited compositional information is available on the labels of most fruit-based supplements, the data in this article will enable consumers to select the required volume of the ten tested supplements to meet suggested recommendations for polyphenol intake to enhance performance (300 mg pre-exercise) and accelerate recovery (1000 mg per day) from prior exercise.

## 1. Introduction

Polyphenols are a diverse group of plant secondary metabolites present in a wide variety of foods, including fruits, vegetables, and cereals [1]. Polyphenols can be divided into four main groups, the phenolic acids, flavonoids, stilbenes, and lignins based on the number of phenol rings they contain and the structural elements that link the rings together [2]. Polyphenol supplementation has been purported to enhance exercise performance [3] and accelerate recovery from exercise-induced muscle damage (EIMD) [4], although the results of all studies have not been positive. The mechanisms underpinning the potential beneficial effects are yet to be fully elucidated, but could be related to their antioxidant, vasodilatory, and anti-inflammatory actions [5].

Tart cherries [6], pomegranates [7], blackcurrants [8], and blueberries [9] have attracted research interest because of their high content of polyphenols, especially anthocyanins (a subclass of the flavonoid group). The results of studies investigating the effects of these fruits on performance and recovery have not been entirely consistent. Some disagreement between studies probably reflects differences in study design, but possible variation in the dose of polyphenols supplied by different fruit supplements could also be a contributing factor. For example, Connolly et al. [10] reported that consumption of a tart cherry/apple juice blend supplying 1200 mg of polyphenols for 4 days prior to and 4 days following 2 sets of 20 maximal eccentric contractions of the elbow flexors enhanced recovery of muscle strength. In contrast, Levers et al. [11] reported that a lower intake of 480 mg/d of Montmorency tart cherry powder for 7 d before and 2 days after 10 × 10 back squats failed to accelerate recovery of muscle strength.

Attempts to disentangle the effect of study design from differences in quantity of polyphenols supplied have in some cases been hindered by omission of the composition of the supplements being investigated. One brand of Montmorency tart cherry concentrate has consistently been shown to enhance recovery from EIMD [12–15], whereas an alternate brand of Montmorency tart cherry failed to accelerate recovery from muscle damage incurred during soccer and rugby matches [16,17]. With no data available on the polyphenolic content of the alternate brand, it is impossible to conclude whether differences in study design or quantity of polyphenols supplied were responsible for the disagreements between these studies.

A review of fruit-derived polyphenols proposed that supplementation with > 1000 mg of polyphenols per day for 3 or more days prior to and following exercise was necessary to accelerate recovery from EIMD [5]. To enhance endurance and repeated sprint performance, it was stated that an intake of ~300 mg of polyphenols an hour before exercise was sufficient. To enable an athlete to select the correct dose of a polyphenol-rich fruit supplement to enhance performance or recovery, it is critical to know the quantity of polyphenols present per serving. For many commercial polyphenol-rich fruit supplements such as pomegranate, Montmorency cherry, blueberry, and blackcurrant, none or only limited data are available to the consumer. Therefore, the primary aim of this study was to produce a compendium of information on the polyphenolic content of a range of polyphenol-rich fruit supplements that are commercially available and commonly used by recreationally active and athletic populations.

## 2. Methods

### Polyphenol-rich fruit supplements

A range of supplements (n = 10) containing cherry, pomegranate, blueberry, or blackcurrant were analyzed in this study (Table 1). All supplements were purchased directly from the manufacturers’ websites except one (POM Wonderful pomegranate juice, which was purchased from a local supermarket). We did not disclose to the manufacturers that we were analyzing the products for a study. Supplements were stored at 4°C prior to analysis and analyzed before their best before dates.

**Table 1.** Details of the polyphenol-rich fruit supplements analyzed.

Supplement	Serving Size	Information from label or website
Active Edge Cherry Concentrate Bottle (948 ml)	30 ml	320 mg of anthocyanins per serving
Active Edge Cherry Concentrate Sachet Informed Sport Certified	30 ml	320 mg of anthocyanins per serving
Active Edge Cherry Capsule	2 capsules (870 mg)	85.2 mg of anthocyanins per serving 154.3 mg of total phenols per serving
Active Edge Blueberry Concentrate (473 ml)	30 ml	378 mg of anthocyanins per serving
Active Edge Pomegranate Concentrate (473 ml)	30 ml	No information available
Healthspan Elite Sour Concentrate Cherry Sachet Informed Sport Certified	30 ml	No information available
Science in Sport Rego Cherry Concentrate Sachet Informed Sport Certified	30 ml	No information available
Pro Athlete Supplementation Cherry Bomb Sachet Informed Sport Certified	40 ml	No information available
POM Wonderful Pomegranate Juice Bottle (710 ml)	250 ml	No information available
CurraNZ New Zealand Blackcurrant Capsule Informed Sport Certified	300 mg (1 capsule)	ORAC: 1946 µmol of Trolox equivalents per capsule 105 mg of anthocyanins per capsule

### Extraction processes for tart cherry capsules

Prior to measuring total phenols and antioxidant capacity in the Active Edge tart cherry capsules, the following extraction process was used [18]. Briefly, the tart cherry powder was added to a solution of methanol/water (80:20 v/v) and shaken overnight in the dark at 5°C. The resulting solution was sonicated for 10 min at 25°C and mixed on a whirl mixer for 30 seconds before analysis. Anthocyanins were extracted from the capsules using the same method except that the extraction solvent was methanol/water/acetic acid (85:15:0.5 v/v) [18].

### Total phenol content

The total phenol content of three samples of each supplement was measured in duplicate using the Folin–Ciocalteu colorimetric assay [19]. Gallic acid standards were prepared in distilled water over the range of 0–100 mg/L. All supplements were diluted in distilled water before analysis to ensure that absorbances fell within the standard curve. A 0.5 ml sample of diluted supplement or standard was added to a test tube containing 2.5 ml of 10% Folin–Ciocalteu reagent, and tubes were mixed. Within 30 sec to 8 min, 2 ml of sodium carbonate (7.5% w/w) was added, and each tube was mixed and incubated for 150 min at 24°C (LTE OP100-UF Oven, LTE Scientific, UK). After incubation, absorbance of each sample was measured at 765 nm (Jenway 315 spectrophotometer, Cole-Palmer, Staffordshire, UK) against a reagent blank. The total phenol content was determined from the standard curve by linear regression and expressed as gallic acid equivalents (GAE) per serving.

### Total anthocyanin content

The total anthocyanin content of three samples of each supplement was determined in duplicates using the pH differential method [20]. Two dilutions of each sample were made, one in 0.025 M potassium chloride (pH 1.0) and the other in 0.4 M sodium acetate (pH 4.5). These were incubated for 20 min at room temperature. Absorbance of the test supplements in the pH 1.0 and pH 4.5 solutions was read at 520 nm and 700 nm (Jenway 315) against a blank of distilled water. Total anthocyanins were calculated using the following equation and expressed as cyanidin-3-glucoside equivalents per serving:

Cyanidin-3-glucoside equivalents, mg/L =

$$\frac{A\times MW\times DF\times 10^{3}}{\varepsilon \times l}$$

where A is the absorbance difference calculated as (A520nm – A700nm) pH 1.0 – (A520nm – A700nm) pH 4.5;

MW (molecular weight) is 449.2 g/mol for cyanidin-3-glucoside (cyd-3-glu);

DF is the dilution factor;

l is the path length in cm;

ε is 26 900, the molar extinction coefficient in L x mol^–1^ x cm^–1^ for cyd-3-glu; and 10^3^ is the factor for conversion from g to mg.

### Ferric reducing/antioxidant power (FRAP) assay

The FRAP assay as described by Benzie and Strain [21] was used to determine the antioxidant capacity of each supplement. FRAP measures the ability of a sample to reduce a ferric-2,4,6-tripyridyl-s-triazine (TPTZ) complex to the ferrous form and is based on single electron transfer [21]. FeSO_4_.7H_2_O standards were prepared in distilled water (0–2000 µM). Three samples of each supplement were diluted in distilled water to ensure that absorbance fell within the standard curve. A 33 µl sample/standard was added to 1 ml of FRAP reagent (300 mM sodium acetate (pH 3.6), 10 mM TPTZ (in 40 mM hydrochloric acid), and 20 mM FeCl_3_∙6H_2_0 in a 10:1:1 ratio) and mixed. After 4 min, absorbance was measured at 593 nm (Jenway 315 spectrophotometer) against a reagent blank. FRAP was calculated from the standard curve using linear regression and expressed as µmol of Fe^2+^ equivalents per serving. Each sample was measured in duplicate.

### Total oxygen radical absorbance capacity (ORAC)

The ORAC assay determines the ability of the antioxidants present in a sample to inhibit peroxyl radicals generated by 2,2´-azobis(2-amidinopropane)dihydrochloride (AAPH) in a reaction mixture and is based on hydrogen atom transfer [22]. ORAC was determined as described by Huang et al. [22], with minor modifications. Briefly, Trolox (6-hydroxy-2,5,7,8-tetramethylchromane-2 carboxylic acid) standards (0–100 µM) were prepared in 75 mM phosphate buffer (75 mM K2HPO4 + 75 mM NaOH2PO4, pH 7.4). Samples were diluted in phosphate buffer to fall within the linear range of the assay. The assay was conducted in black-walled 96-well polystyrene plates. To create a high thermal mass, the exterior wells of the 96-well plates were filled with 300 µl of water, and thus, the experimental wells were limited to 60 inner wells. Fluorescein disodium (150 µl; 4 × 10^−6^ mM) was added to each experimental well, followed by 50 µl of standard or sample. The plate was then incubated for 30 min at 37°C in a BioTek Synergy HT Multi-Detection Microplate Reader (BioTek Instruments Inc. Winooski, VT, USA). The ORAC reaction was initiated with the addition of 25 µl of 153 mM AAPH followed by shaking for 1 min at 400 rpm on a plate shaker (ELMI Sky Line DTS4, Newbury Park, CA, USA). Fluorescence (excitation wavelength 485 nm, emission wavelength 530 nm) was recorded every min for 60 min. The net area under the curves (AUC) for the standards and samples was calculated. ORAC values of the samples were calculated by regression of the net AUC for each sample against the Trolox standard curve and are expressed as µmol of Trolox equivalents per serving. Three samples of each supplement were measured in triplicate.

## 3. Results

Table 2 summarizes the content of total phenols and anthocyanins and *in* *vitro* antioxidant capacity of the fruit-based supplements per the recommended serving sizes and per ml. For all measures, there was considerable variability across the supplements. The order of total phenolics per serving size for the supplements from highest to lowest was POM Wonderful Pomegranate Juice > PAS Cherry Bomb > Active Edge Blueberry > Active Edge Pomegranate > SIS Rego Cherry Juice > Healthspan Elite Sour Cherry > Active Edge Cherry Bottle > Active Edge Cherry Sachet > Curra NZ > Active Edge Cherry Capsule, with an 83-fold difference between the highest and lowest supplement.

**Table 2.** Content of total phenols and anthocyanins and the *in vitro* antioxidant capacity per serving of polyphenol-rich fruit supplements.

Polyphenol-rich Product	Total Phenols (mg/GAE)		Total Anthocyanins (mg/cyd-3-glu)		FRAP (µmol of Fe^2+^ equivalents per serving)		ORAC (µmol of TE per serving)	
	Per serving	Per ml	Per serving	Per ml	Per serving	Per ml	Per serving	Per ml
POM Wonderful 250 ml glass	1007 ± 159	4.0	40.5 ± 0.5	0.16	14,320 ± 573	57.3	5938 ± 684	23.8
PAS Cherry Bomb 40 ml	591 ± 23	14.8	23.7 ± 3.8	0.59	4489 ± 300	112	8362 ± 376	209
Active Edge Blueberry 30 ml	432 ± 55	14.4	7.6 ± 1.8	0.25	4260 ± 1134	142	6670 ± 553	222
Active Edge Pomegranate 30 ml	391 ± 27	13.0	6.8 ± 0.6	0.23	6464 ± 1472	216	2897 ± 299	96.6
SIS Rego Cherry Juice 30 ml	321 ± 18	10.7	14.3 ± 1.0	0.48	2966 ± 151	98.9	10,072 ± 484	336
Healthspan Elite Sour Cherry 30 ml	247 ± 20	8.2	5.5 ± 0.6	0.18	2624 ± 219	87.5	8917 ± 299	297
Active Edge Cherry Bottle 30 ml	239 ± 18	8.0	4.0 ± 0.4	0.13	3252 ± 458	108	7408 ± 926	247
Active Edge Cherry Sachet 30 ml	208 ± 8	7.0	1.5 ± 0.7	0.05	1724 ± 80	57.5	6015 ± 174	201
CurraNZ 1 × 300 mg capsule	169 ± 13	*562 per g	73.2 ± 5.6	*244	1179 ± 134	*3928	1160 ± 85	*3868
Active Edge Capsule 2 × 435 mg capsules	12 ± 1	*13.8 per g	0.2 ± 0.1	*0.22	72 ± 6	*82.3	150 ± 16	*173

Values are presented as means ± SD; 3 samples of each supplement were analyzed in duplicate

GAE: Gallic acid equivalents

TE: Trolox equivalents

*: per g of powder

The overall order for total anthocyanin content per serving was Curra NZ > POM Wonderful Pomegranate Juice > PAS Cherry Bomb > SIS Rego Cherry Juice > Active Edge Blueberry > Active Edge Pomegranate > Healthspan Elite Sour Cherry > Active Edge Cherry Bottle > Active Edge Cherry Sachet > Active Edge Cherry Capsule (Table 2). There was a 385-fold difference between the supplement with the highest content of anthocyanins (1 x CurraNZ blackcurrant capsule) and the lowest (2 x Active Edge Cherry capsules).

Table 3 compares our measured values for total anthocyanin and ORAC content vs. manufacturer/supplement label values for the five supplements where the information was available. Our data for the total anthocyanin content for one Curra NZ blackcurrant capsule were 70% of the value reported by the manufacturer. The anthocyanin content of the other supplements was much lower than the label value and ranged from 0.2 to 2%. The measured ORAC values for the supplements ranged from 15 to 90% of the values reported by the manufacturers.

**Table 3.** Comparison of anthocyanin content and ORAC between the analyzed and manufacturers’ values (where available).

	Anthocyanins (mg per serving)		ORAC (µmol of Trolox equivalents per serving)	
Product	Manufacturer’s Value	Analyzed Value (% of manufacturer’s value)	Manufacturer’s Value	Analyzed Value (% of manufacturer’s value)
Active Edge Cherry Bottle (30 ml serving)	320	4.0 ± 0.4 (1.3%)	8260	7408 ± 926 (90%)
Active Edge Cherry Sachet (30 ml serving)	320	1.5 ± 0.7 (0.5%)	8260	6015 ± 174 (73%)
Active Edge Cherry Capsule (2 capsules)	85.6	0.2 ± 0.1 (0.2%)	986	150 ± 16 (15%)
Active Edge Blueberry (30 ml serving)	378	7.6 ± 1.8 (2.0%)	No data available	Comparison not possible
Curra NZ (1 capsule)	105	73.2 ± 5.6 (70%)	1946	1160 ± 85 (60%)

Values are presented as means ± SD; 3 samples of each supplement were analyzed in duplicate

ORAC – Oxygen Radical Absorbance Capacity

Table 4 provides information on the required number of servings or volume of fruit concentrate required to meet recommendations for enhancing performance (~300 mg of polyphenols) and recovery from EIMD (> 1000 mg of polyphenols). According to our data, there is a substantial difference in the number of servings of each supplement required to achieve these recommendations.

**Table 4.** Minimum number of servings of each polyphenol-rich product to supply approximately 300 mg (for performance) or 1000 mg (for recovery) of polyphenols according to our analyses.

Single serving size of each product	Total Phenols (mg/GAE)	Approximate number of servings required for performance (~ 300 mg)	Approximate number of servings required for recovery (> 1000 mg)
	*Per serving*	*Serving size*	*Serving size*
POM Wonderful 250 ml glass	1007 ± 159	75 ml (~ ⅓ of a glass)	1 glass
PAS Cherry Bomb 40 ml sachet	591 ± 23	½ of a sachet	2 sachets
Active Edge Blueberry 30 ml of bottle	432 ± 55	21 ml	69 ml
Active Edge Pomegranate 30 ml of bottle	391 ± 27	23 ml	77 ml
SIS Rego Cherry Juice 30 ml sachet	321 ± 18	1 sachet	3 sachets
Healthspan Elite Sour Cherry 30 ml sachet	247 ± 20	1 ⅕ sachets	4 sachets
Active Edge Cherry 30 ml of bottle	239 ± 18	38 ml	126 ml
Active Edge Cherry 30 ml sachet	208 ± 8	1 ½ sachets	5 sachets
CurraNZ 1 x 300 mg capsule	169 ± 13	2 capsules	6 capsules
Active Edge Capsule 2 x 435 mg capsules	12 ± 1	50 capsules	166 capsules

## 4. Discussion

Polyphenol-rich fruit supplements have become popular among recreational and elite athletes. A comprehensive review of polyphenols and exercise concluded that a dose of 300 mg of polyphenols before exercise enhances performance and 1000 mg per day for several days accelerates recovery [5]. However, it is difficult for athletes, coaches, and sport nutritionists to select a particular polyphenol-rich fruit supplement to meet these recommendations, because only limited compositional information is available on the labels of many supplements. In this study, we found that 10 commercial polyphenol-rich fruit supplements varied substantially in the quantity of polyphenols and anthocyanins they contained per serving. While it would be expected that supplements made from different types of fruit would vary in their composition, we also found that different brands of tart cherry varied by approximately three-fold in their content of total phenols and 15-fold in their content of anthocyanins. Moreover, we found that the content of total phenols and anthocyanins in the fruit supplements was lower than manufacturer’s values where available.

Comparison of nine of the supplements (excluding one with exceptionally low phenol and anthocyanin content) revealed an approximately 6-fold difference in the content of total phenols and 126-fold difference in the content of intact anthocyanins per serving. There were also large variations in the antioxidant capacity of the various fruit supplements when assessed by two *in* *vitro* assays, FRAP (12-fold variation) and ORAC (9-fold variation). It is uncertain what components of polyphenol-rich fruit supplements are responsible for enhancing performance and recovery, but it is possible that the efficacy of each product may vary according to the quantity of polyphenols and anthocyanins supplied per typical serving.

Montmorency tart cherry concentrates have been the most frequently investigated polyphenol-rich fruit supplement proposed to accelerate recovery from EIMD [4]. In a recent review of fruit-derived polyphenols and exercise, it was stated that 30 mL of Montmorency tart cherry concentrate supplies 600 mg of total phenols [5]. This value presumably helped to inform the recommendation that 1000 mg/d of total phenols are needed to accelerate recovery from EIMD. However, the origin of the 600 mg value for total phenols is unclear because the studies cited in the review [12–14,23,24] did not measure nor report a value for total phenols. In our analyses, we found that 30 mL of the brand of tart cherry concentrate used in these studies only contained approximately 250 mg of total phenols. Across the five tart cherry juice concentrates analyzed, there was an approximately three-fold difference in the content of total phenols, with all containing less than 600 mg. The other supplements analyzed also varied substantially in total phenol content per serving. This suggests the need for researchers to measure and report the total phenol content of the fruit supplements they administer to allow accurate interpretation of their results, so definitive recommendations for effective doses can be made to sport nutritionists and athletes.

It has been proposed that anthocyanins may contribute to the beneficial effects of polyphenol-rich fruit supplements because they have been shown to promote vasodilation [25], induce antioxidant defenses [26], and exert anti-inflammatory effects [27]. Therefore, knowing the content of anthocyanins in a supplement may inform decisions on their use. Using the pH differential method, servings of most of the fruit-based supplements were found to supply a low number of anthocyanins, except for New Zealand blackcurrant capsules and to a lesser extent POM Wonderful pomegranate juice. Our values for total anthocyanin content are consistent with those available in the literature for tart cherry concentrate (1.5 to 23.7 mg per serving v 2 [28] to 15.6 mg per serving) [29] and pomegranate juice (40.5 mg v 38.4 mg per 250 mL) [30]. Values from the manufacturers for total anthocyanins were available for 5 of the 10 supplements included in this study. However, we found much lower levels of anthocyanins in these supplements. Anthocyanins are susceptible to degradation during transportation, storage, and processing [31]. All supplements were refrigerated after purchase, but they were delivered unrefrigerated, and we have no details of the storage conditions before purchase. This could partly explain the discrepancy between our values and manufacturers’ values because the pH differential method does not measure degraded anthocyanins. Discrepancies could also partly reflect the use of a different method by manufacturers to measure total anthocyanins, but we were unable to confirm this. The low amounts of anthocyanins we found in most of the polyphenol-rich fruit products indicate that anthocyanin content may not be the major determinant of whether a fruit supplement accelerates recovery from prior exercise or enhances performance.

We used two *in* *vitro* assays of total antioxidant capacity to characterize the different fruit supplements, ORAC and FRAP. Both assays have been used in the exercise literature to characterize various foods and supplements [9,32,33]. Our ORAC results for Active Edge tart cherry and CurranNZ blackcurrant powder were broadly consistent with manufacturers’ values and our POM Wonderful values with the literature [34], whereas, for Active Edge tart cherry capsules, our results were only 15% of the manufacturer’s values. FRAP values have not been reported by manufacturers of the supplements we analyzed; however, our FRAP value for POM Wonderful was consistent with that in the study by Borges et al. [30]. Based on our data, nine of the ten supplements would be classified as high in antioxidant capacity per serving when compared to data of 1120 food samples from the United States Department of Agriculture National Food and Nutrient Analysis Programme [35]. However, the *in vitro* assays we used measured the content of antioxidant/reducing compounds within a product and do not necessarily indicate whether the product will exert an antioxidant effect after consumption. This may be particularly pertinent to polyphenol supplements because polyphenols tend to be poorly absorbed, undergo metabolism to other compounds after consumption, and are unlikely to reach sufficient concentrations *in vivo* (except in the gut lumen) to act as direct antioxidants [36,37]. Indeed, it is likely that any systemic antioxidant activity of ingested polyphenols reflects the ability of their metabolites to activate endogenous antioxidant enzymes [37]. Consequently, the high *in vitro* antioxidant capacity of fruit supplements reported here needs to be interpreted with caution.

### Limitations and future directions

Our analyses were limited to polyphenol-rich fruit supplements that were widely available and commonly used by sport nutritionists (unpublished observations) and athletes within the UK so may not include certain supplements popular outside of the UK. The supplements were purchased directly from the manufacturers’ websites (except for POM Wonderful, which was purchased from a supermarket) and kept refrigerated and protected from light upon delivery, but we have no details of prior storage conditions (i.e. duration, temperature, and light exposure). This might have led to variable degradation of polyphenolic compounds and may explain some of the variability we found between the fruit-based supplements. However, the consumers of these products would also be exposed to this potential source of variability in composition when purchasing the product. We could only detect low levels of total phenolics and anthocyanins in tart cherry capsules. The low values may reflect failure to extract all the phenolic compounds, because the powder in the capsules did not readily dissolve in water and a range of buffers despite the use of agitation and sonication techniques. Nevertheless, our values are consistent with those reported by Kirakosyan et al. [18] for Montmorency tart cherry powder. It is possible that more polyphenols are extracted from tart cherry capsules during digestion because supplementation with these capsules has been shown to improve performance [38].

The primary purpose of this study was to provide an overview of the total phenolic and anthocyanin content of fruit supplements. Future work determining the profile of individual polyphenols present in each supplement could further enhance understanding of the active components present and explain some of the inconsistencies in the performance and recovery literature. A survey we conducted with nutrition practitioners working in professional soccer (unpublished observations) found that most store polyphenol-rich fruit supplements for protracted periods at room temperature before use, which may lead to degradation of polyphenols. Therefore, there is a need for future studies to investigate the effect of storage conditions on the stability of polyphenols in fruit supplements and to identify any polyphenolic degradation products. It is likely that different batches of the same fruit supplement vary in polyphenol content, which should also be explored.

### Practical applications

As it would be expected, we found that a typical serving of each polyphenol-rich fruit supplement contained different quantities of anthocyanins and polyphenols. Therefore, to reach recommended target intakes (i.e. 1000 mg to promote recovery [5]), athletes will need to adjust the number of servings of their chosen supplement according to its content of polyphenols (see Table 4). The purpose of this study was not to promote individual supplements but to provide sport nutritionists, coaches, and athletes with the required information to select the required volume of a supplement to suit their performance or recovery goals. For example, to reach 1000 mg of total phenols, athletes would need to consume 1 glass of pomegranate juice vs. two to five servings of tart cherry concentrate vs. six capsules of New Zealand Blackcurrant powder.

## 5. Conclusions

In conclusion, the 10 fruit supplements we analyzed varied substantially in their content of total phenols per suggested serving size. Most contained lower levels of intact anthocyanins per serving than expected, apart from CurraNZ blackcurrant powder and POM Wonderful pomegranate juice. While there is a scope for a more comprehensive analysis of the polyphenolic profile of the supplements tested, our findings could be used by athletes, coaches, and sport nutritionists to help guide their use of these supplements. The differences reported here also indicate the need for researchers to clearly characterize the composition of the polyphenol-rich supplements they investigate to aid the interpretation of their results and inform recommendations on effective doses.

## Data Availability

The data used for this study are available from the corresponding author on reasonable request.
